# Feedback function for capillary refilling time measurement device

**DOI:** 10.1186/s13054-019-2570-y

**Published:** 2019-09-03

**Authors:** Masayoshi Shinozaki, Taka-aki Nakada, Rui Kawaguchi, Yuichiro Yoshimura, Toshiya Nakaguchi, Hideaki Haneishi, Shigeto Oda

**Affiliations:** 1 Center for Frontier Medical Engineering, Chiba University, 1-33, Yayoicho, Inage, Chiba, 263-8522 Japan; 2 Department of Emergency and Critical Care Medicine, Chiba University Graduate School of Medicine, 1-8-1 Inohana, Chuo, Chiba, 260-8677 Japan

Capillary refilling time (CRT) is an important indicator of microcirculation [1, 2]. To develop a CRT measurement device, the optimal strength and time for pressing the nail bed were investigated [3]. However, whether examiners can precisely achieve optimal strength and time remains unknown. Thus, the requirements for a CRT measurement device have not yet been fully elucidated. We developed a portable CRT measurement device with a feedback function to achieve optimal strength and time (Fig. 1) and tested a hypothesis that the feedback function is the key for satisfying the measurement conditions of the CRT device.

**Fig. 1:**
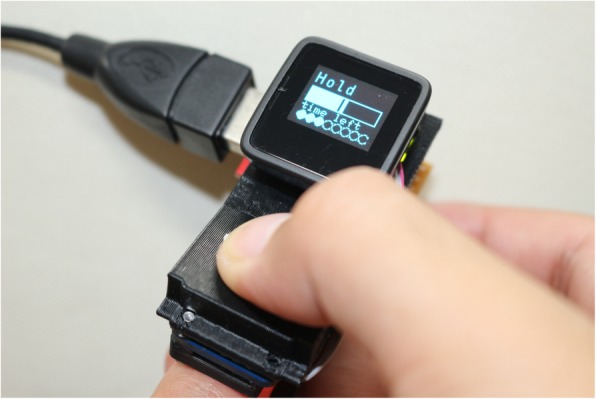
Photograph of the developed device. The feedback function is shown in an OLED display

The CRT was measured by 20 examiners using the developed device with and without a feedback function. According to a previous report [3], the target strength of 5 N and time of 5 s were obtained (Additional file 1). The pressing strength and time during the CRT measurement were evaluated.

A significant difference was found in the pressing strength and time between the CRT measurement using the device with and without a feedback function (strength: *P* < 0.001; time: *P* < 0.01). The feedback function significantly reduced the intra-examiner variance in the pressing strength and time (strength: *P* < 0.001; time: *P* < 0.001) (Fig. 2). In all measurements without the feedback function, 41% of the pressing strength was outside the required strength range. In contrast, in the CRT measurements with the feedback function, 100% of the pressing strength was successfully achieved within the target range. The pressing time with the feedback function achieved the target time in all measurements, whereas 12% of the measurements without the feedback function exhibited insufficient pressing time. In total, 49% of the measurements without the feedback function failed to satisfy the required conditions.

**Fig. 2:**
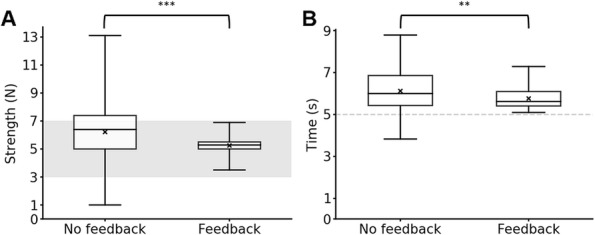
**a**, **b** Pressing strength and time in the CRT measurement using the developed device without and with feedback function. Panel **a** Pressing strength. Panel **b** Pressing time. A significant difference was observed in the pressing strength and time between the CRT measurements using the portable CRT device with and without a feedback function (Mann–Whitney *U* test—strength: *P* < 0.001; time: *P* < 0.01). The feedback function significantly reduced the intra-examiner variance in the pressing strength and time (*F* test—strength: *P* < 0.001; time: *P* < 0.001). The median and minimum and maximum interquartile ranges are shown

Compared with the high failure rate of the CRT measurements without the feedback function, the feedback function, which served as a guide for the pressing strength and time, achieved complete success in fulfilling the required measurement conditions of the CRT measurement using the portable device. The critical issue of the CRT measurement is the intra-observer difference [4]. Personal work experience and training have been suggested to possibly help improve the accuracy of CRT measurements [5]. Evidently, in the present study, the feedback function significantly reduced the intra-examiner variance. Thus, this device would be a smart solution for intra-observer difference regardless of personal work experience and without training. Further development of portable CRT measurement devices with feedback functions may contribute to achieve precise CRT measurements to monitor microcirculation in clinical settings.

## Additional file

Additional file 1: Methods and supplemental data. **Figure E1.** Appearance of the developed device and display of the feedback function. (a) Front image. (b) Side image. (c) Oblique image with a finger. (d)–(h) Display changes in the feedback function. (d) Ready for measurement. (e) The filled range indicates the strength applied to the nail bed, and the vertical line shows the target strength. This display indicates insufficient pressing strength. (f) The display shown after the target strength is applied. The filled circle indicates the time to press, and the blue circle indicates the remaining time to press the nail bed. (g) The display tells the examiner to release the compression. (h) The display is shown after the release of the nail bed, which shows the interval until the next measurement. (PDF 1307 kb)

## Data Availability

The datasets used and analyzed during our study are available from the corresponding author upon reasonable request.
