# Supplementary material for: Feedback function for capillary refilling time measurement device
Source: Crit Care. 2019 Sep 3;23:295. doi: 10.1186/s13054-019-2570-y (PMC6724324; doi:10.1186/s13054-019-2570-y)

Feedback function for capillary refilling time measurement device

Masayoshi Shinozaki, BEng; Taka-aki Nakada, MD, PhD; Rui Kawaguchi, MD; Yuichiro Yoshimura, PhD; Toshiya Nakaguchi, PhD; Hideaki Haneishi, PhD; Shigeto Oda, MD, PhD

Online Data Supplement

1. Methods

1.1 Device

We developed a portable CRT measurement device that detects the change in color of a nail bed caused by manually pressing and releasing the nail bed. We developed a feedback function to achieve optimal strength and time for stable measurement (**Figure E1**).

The color change in the nail bed was measured using a reflecting-type photosensor, which is a color sensor (Adafruit Industries, RGB Color Sensor with IR cut filter, TCS34725, USA), and the illumination source was provided by a white light-emitting diode (LED). An analog–digital converter for the color sensor was set for 24 ms of integration time and for quadruple gain.

The feedback function consisted of a force-sensing resistor (Interlink Electronics Inc., FSR400) and a microcontroller mounted organic LED display (SparkFun Electronics, MicroView). The display illustrated the guide information so that the examiner could press the nail bed to achieve optimal strength and time [**Figures E1(d)–(h)**].

The shape of the device was similar to a clip-type pulse oximeter, and it enabled the examiner to measure the CRT in a simple and practical manner. The outer frame of the device was made of ABS resin using a 3D printer, the size was 59 mm × 21 mm × 50 mm, and the total weight was 31 g.

1.2 Subjects and Study Protocol of the Measurements

Twenty examiners (male =10; female = 10) were enrolled. The CRT was measured at the left index finger using the developed portable CRT device with and without a feedback function. According to a previous report [1], we set the target strength to 5 N and the time to 5 s.

Before the CRT measurement tests, every examiner operated the CRT measurement device once for orientation on the operating procedure. First, the examiner repeatedly measured the CRT with a feedback function for five times at a 5-s interval. Then, the examiner performed

the same measurement under the same process and conditions but without the feedback function. The pressing strength was recorded by the force sensor every 30 ms in the duration from pressing to releasing the nail bed. The pressing time was defined as the duration from the initiation of pressing the nail bed to its release.

1.3 Statistical Analysis

The differences in the measurements between those with and without the feedback groups were analyzed using the Mann–Whitney U test. The intra-examiner variance in the pressing strength and time were analyzed using the F-test. Two-tailed *P* values of less than 0.05 were considered significant. All statistical analyses were performed using Python version 3.7 with SciPy version 1.2.1 and matplotlib version 3.0.3.

REFERENCES

1. Kawaguchi R, Nakada TA, Oshima T, Shinozaki M, Nakaguchi T, Haneishi H, Oda S: Optimal pressing strength and time for capillary refilling time. Crit Care 2019, 23:4.

Figure Legends

Figure E1. Appearance of the developed device and display of the feedback function

(a) Front image

(b) Side image

1. Microcontroller mounted display
2. Microcontroller mounted display USB programmer
3. Force-sensing resistors
4. Color sensor and indenter

(c) Oblique image with a finger

(d)–(h) Display changes in the feedback function

(d) Ready for measurement

(e) The filled range indicates the strength applied to the nail bed, and the vertical line shows the target strength. This display indicates insufficient pressing strength.

(f) The display shown after the target strength is applied. The filled circle indicates the time to press, and the blue circle indicates the remaining time to press the nail bed.

(g) The display tells the examiner to release the compression.

(h) The display is shown after the release of the nail bed, which shows the interval until the next measurement.

Figure E1. Appearance of the developed device

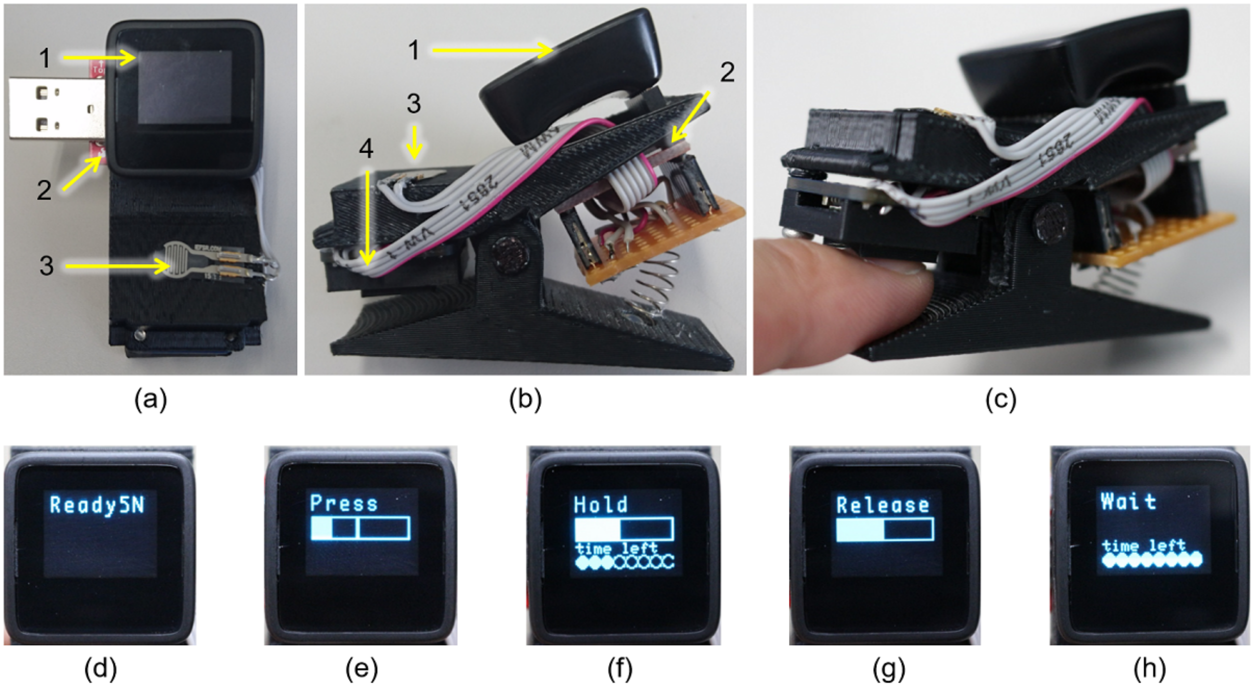

Supplement: Supplementary file 1 — Methods and supplemental data. Figure E1. Appearance of the developed device and display of the feedback function. (a) Front image. (b) Side image. (c) Oblique image with a finger. (d)–(h) Display changes in the feedback function. (d) Ready for measurement. (e) The filled range indicates the strength applied to the nail bed, and the vertical line shows the target strength. This display indicates insufficient pressing strength. (f) The display shown after the target strength is applied. The filled circle indicates the time to press, and the blue circle indicates the remaining time to press the nail bed. (g) The display tells the examiner to release the compression. (h) The display is shown after the release of the nail bed, which shows the interval until the next measurement. (PDF 1307 kb) [file 13054_2019_2570_MOESM1_ESM.pdf]
